# Understanding Genomic Landscapes of Differentiation in Round-Tailed Horned Lizards (*Phrynosoma modestum*)

**DOI:** 10.1093/gbe/evag217

**Published:** 2026-08-28

**Authors:** Julia Amoroso, Keaka Farleigh, Erika Crispo, Christopher Blair

**Affiliations:** Biology PhD Program, CUNY Graduate Center, New York, NY 10016, USA; Department of Invertebrate Zoology, American Museum of Natural History, New York, NY, USA; Department of Biology, University of Virginia, Charlottesville, VA 22904, USA; Department of Biology, Pace University, New York, NY 10038, USA; Biology PhD Program, CUNY Graduate Center, New York, NY 10016, USA; Department of Biological Sciences, New York City College of Technology, The City University of New York, Brooklyn, NY 11201, USA

**Keywords:** *Phrynosoma modestum*, islands of divergence, allopatry, speciation

## Abstract

Population divergence is promoted by divergent selection and inhibited by gene flow, but the mechanisms of and relationship between these two processes remain poorly understood. Developing a well-informed hypothesis of the selective pressures underlying divergence in a natural population requires a thorough understanding of both species structure and demographic history. In this study, we assess whole-genome sequences of round-tailed horned lizards (*Phrynosoma modestum*) from throughout the species range and combine phylogenetic analyses with genomic landscape scans to understand how current genetic diversity has been influenced by demographic histories and evolutionary pressures. Maximum likelihood (ML) phylogenetic analysis supports two lineages within the species, corresponding to a North/South population divide that developed around 7 million years ago (Ma) and displays little migration. However, intermediate genealogical divergence index values between the two lineages ultimately leave us unable to recommend a full taxonomic distinction. Genome-wide scans of population genetic statistics identified islands of divergence exhibiting differentiation patterns linked to models of reproductive isolation and within-population selection. Significantly negative values of Tajima's *D* and positive selection statistics in these islands offer support for selection acting on *P. modestum*, but patterns may also stem from recent population expansions. We posit that selection within populations has played a large role in shaping genomic divergence across the species' range. Taken together, our results provide perspective into how variable selective pressures shape the genomics of two divergent populations currently maintaining species integrity, despite significant signatures of geographic structure and divergence.

Significance Evaluating where evolutionary forces such as selection and gene flow act on a genome furthers our understanding of how populations within the same species begin to diverge from one another. To address this question, we scanned whole-genome samples of round-tailed horned lizards and modeled their evolutionary history, identifying regions where genetic content differed significantly between populations of the species. Our findings provide additional context on where peaks of differentiation can develop between two distinct lineages and advance an understanding of the early stages of population divergence.

## Introduction

Understanding the drivers of intraspecific variation is key to understanding speciation and the origins of biodiversity (Hahn 2019). In natural populations, intraspecific variation is generated and influenced by evolutionary processes closely linked with population divergence. Genetic drift and divergent selection increase divergence between populations and, over time, result in the evolution of reproductive isolation (Kimura 1968; King and Jukes 1969; Nei et al. 1983). Contrastingly, gene flow decreases divergence and inhibits the evolution of reproductive isolation (Mayr 1963; Wu 2001; Coyne and Orr 2004; Nosil 2008). Yet, our understanding of these processes, the genomic regions they influence, and the genes they involve remains incomplete.

Intraspecific variation is often characterized as a heterogeneous genomic landscape of diversity and differentiation, with peaks and troughs driven by neutral (eg. genetic drift) and selective processes (eg. divergent selection) that differentially impact genomic regions (Burri et al. 2015; Wolf and Ellegren 2017; Chase et al. 2021). Early studies of these differentiation peaks referred to them as “speciation islands” because they were thought to contain loci involved in incipient reproductive isolation, although we now understand that these peaks can be driven by any process that reduces diversity (Turner et al. 2005; Cruickshank and Hahn 2014).

**Fig. 1.**
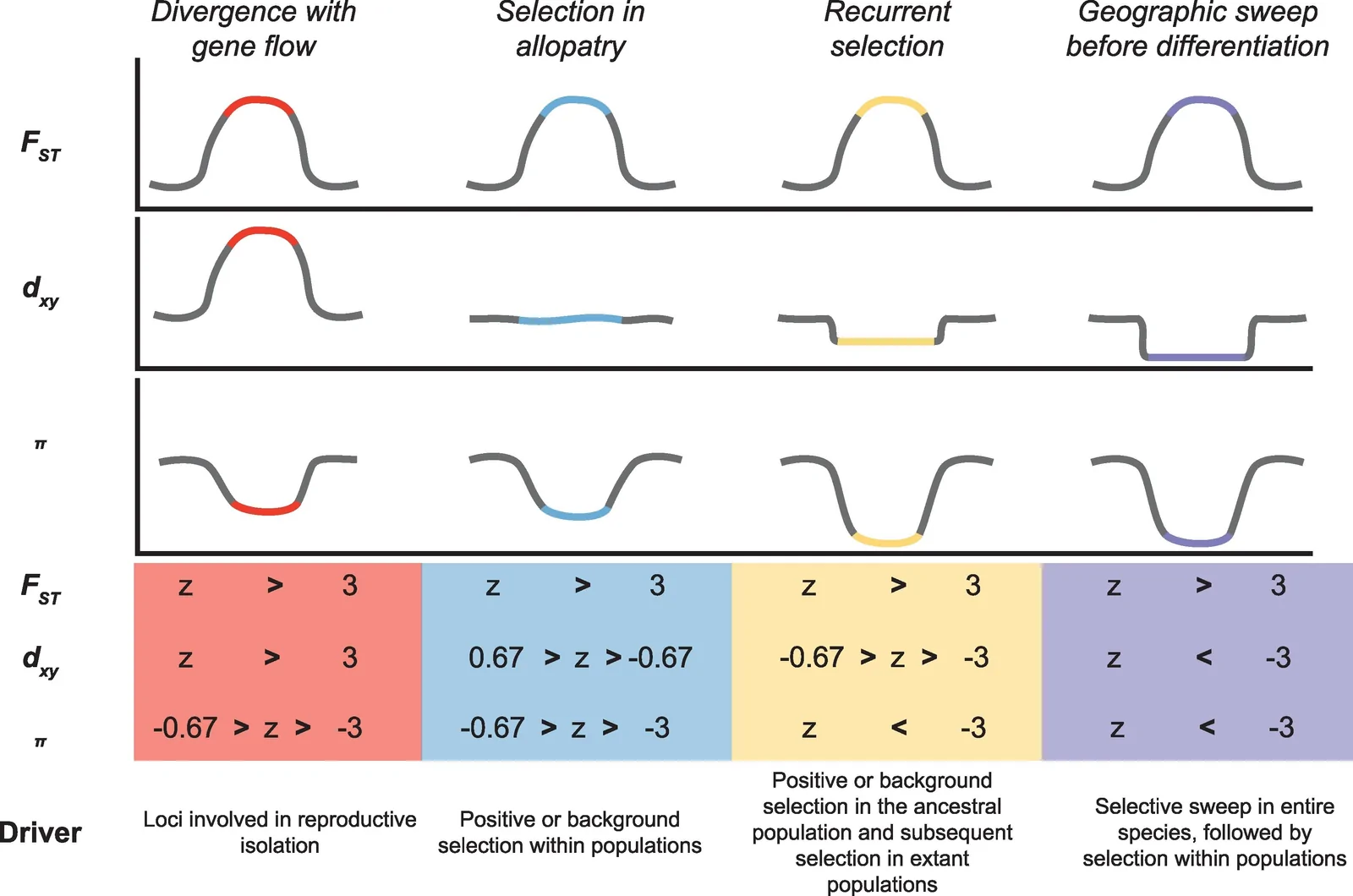
Conceptual visualization of different scenarios that form genomic divergence islands. The illustrations show the relationship between the genomic divergence island (colored region) and the genomic background (gray). The thresholds chosen by the authors and used to identify candidate windows are in the colored boxes, and the source of selection in each scenario is listed at the bottom. A z-score between 0.67 and −0.67 represents ∼50% of windows, a z-score between −0.67 and −3 represents ∼25% of windows, and a z-score greater than 3 represents 0.13% of windows. These scenarios and the sources of their selection were redrawn from Irwin et al. (2018).

Key to our current understanding of how population divergence develops in natural populations has been hypothesizing models of divergence to categorize islands of differentiation based on recognizable genetic signatures characteristic of the evolutionary history that shaped these divergent regions (Irwin et al. 2018; Shang et al. 2023). Pairing F_ST_ with standardized metrics of absolute sequence divergence between populations (*d_xy_*) and nucleotide diversity within populations (π) allows biologists to determine if F_ST_ peaks may be driven by neutral or selective processes and make predictions about the type of selection that acted on divergent genomic regions (Turner et al. 2005; Cruickshank and Hahn 2014; Irwin et al. 2018). Irwin et al. (2018) proposed four models to understand the type of selection that led to the formation of genomic regions of divergence. The first model, divergence with gene flow, suggests that an F_ST_ peak is due to a locus involved in reproductive isolation that has been targeted by divergent selection. Increased F_ST_ is driven by increased *d_xy_*, which reflects a deeper coalescence at that locus relative to the rest of the genome and decreased π (Delmore et al. 2018; Irwin et al. 2018). The second model, selection in allopatry (Fig. 1), relies on within-population selection to explain an F_ST_ peak: increased F_ST_ is driven by decreased π, which reflects faster lineage sorting (Delmore et al. 2018) and more recent coalescence within a population. This assumes that either the selective pressures or descendant population responses to these pressures are distinct, or F_ST_ would not be increased. *d_xy_* is not influenced by within-population selection since it reflects the coalescence between populations, and these loci are not involved in reproductive isolation. As a result, multiple models can be supported within the same genome since different forms of selection can target different parts of the genome; for example, loci involved only in reproductive isolation will exhibit distinct signatures from those involved only in within-population selection. The remaining two models are variants of the selection in allopatry model (Delmore et al. 2018) and involve selection in the ancestral population and a geographic sweep (spread of an advantageous allele across space) followed by selection within populations (Fig. 1). These models are not mutually exclusive, however, as peaks of differentiation may be driven by multiple processes (Schield et al. 2025). Therefore, we must treat these models as suggestive and likely oversimplifications of reality and should pair them with additional tests to identify evidence of selection and the drivers of divergence.

Correctly analyzing the signal of islands of divergence can be clouded by the effects of both genetic architecture and long-term evolutionary dynamics (Nosil et al. 2009; Cruickshank and Hahn 2014; Irwin et al. 2018). Variation in recombination rate across the genome, for example, can play an outsized role in shaping a heterogeneous genomic landscape: low rates of recombination between putative barrier loci and neutral sites can increase the buildup of divergence through linked selection, whereas high rates of recombination can break up linkage between combinations of advantageous alleles and reduce differentiation between lineages (Abbott et al. 2013; De Jode et al. 2023, Nachman 2002). Therefore, directly estimating recombination rate can further contextualize the evolutionary history inferred from windows of divergence (Noor and Bennett 2009; Martin et al. 2013; Renaut et al. 2013; Han et al. 2017). Additionally, historical introgression events between ancestral lineages leave similar genomic signatures to currently ongoing gene flow, underscoring the importance of considering the species' broader evolutionary history when attempting to detect islands of divergence (Pavón-Vázquez et al. 2024).

Linking models of selective pressures in islands of divergence with a phylogeographic examination of species history and structure will deepen our understanding of how and why selective pressures develop in natural populations (Brown et al. 2016; Ravinet et al. 2017; Edwards et al. 2022; Blair et al. 2026). Phrynosomatid lizards serve as an ideal system to connect models of genomic divergence with broader-scale evolutionary histories, as recent genomic work in the *Phrynosoma* (horned lizard) genus has largely resolved the phylogenetic relationships between members of the group (Leaché and Linkem 2015; Leaché et al. 2021). Within *Phrynosoma*, species-level evolutionary histories and population structure have been examined for many species, yet round-tailed horned lizards have been notably excluded (Farleigh et al. 2021; Finger et al. 2022; Gottscho et al. 2024). The round-tailed horned lizard (*Phrynosoma modestum*) is a member of the *Doliosaurus* clade of *Phrynosoma* and diverged from its sister species, *Phrynosoma platyrhinos* and *Phrynosoma goodei*, roughly 15 million years ago (Ma; Leaché and McGuire 2006; Leaché and Linkem 2015). Species distribution estimates for *P. modestum* range throughout the arid regions of southwestern North America, spanning the mesa region of southeastern Arizona into western and southern Texas and extending into northern Mexico (Weese 1917). Numerous biogeographic features divide this range, including the Rocky Mountains to the north; multiple water features, including the Rio Grande and Pecos; and rapid biotic changes to the south (Bundy and Neess 1958; Pavón-Vázquez et al. 2024; Blair et al. 2026). The heterogeneous geographic and climatic conditions of this region have made it a focus of other population genetic and phylogeographic studies that seek to understand lineage divergence in response to long-term evolutionary processes, offering a rich literature in which to situate the biogeographic history of *P. modestum* (Schield et al. 2018; Myers et al. 2019; Provost et al. 2021).

Despite its broad ecological distribution and intersection with multiple biogeographic barriers, ecological and genomic work on *P. modestum* has been sparse, likely due to its small size and cryptic coloration (Whiting and Dixon 1996). However, in an experimental behavioral study, members of the species presented a smaller home range than expected under random conditions and displayed phenotypic variation in dorsal color (Munger 1984). While this presents compelling evidence for the possibility of population differentiation across the species range, further investigation should verify if and how genetic divergence has evolved across the range of the species.

Here, we assess genomic variation and divergence across the native range of *P. modestum* to explore the selective pressures and demographic histories shaping current population diversity. The observation of phenotypic variation, coupled with significant geographic barriers across the species range and evidence for limited home range sizes, led us to hypothesize that *P. modestum* will display population divergence across its genome due to past demographic effects and selective pressures linked to reproductive isolation and within-population selection. By analyzing multilocus and SNP datasets based on whole-genome sequences, we carry out population structure analyses and reconstruct the species' evolutionary history using multiple tree-based methods. To assess selective pressures associated with genetic divergence within *P. modestum*, we analyze signatures of each of four models of islands of divergence, directly test for selective sweeps, evaluate recombination rate within divergence windows, and perform gene ontology analyses for genes in proximity to divergent genomic windows. We then discuss how these explicit tests of species-level evolutionary history interact with selective histories inferred directly and indirectly by population genetic statistics.

## Results

### SNP Filtering

After sequencing, we obtained a median of 154,081,250 reads per sample. An average of 139,569,010 reads mapped to the *P. platyrhinos* reference genome, representing 93.57% of the genome with an average depth of 10.04 across samples (Table S2). After initial variant calling and filtering performed by Novogene following GATK's best practices workflow (Van der Auwera and O’Conner 2020), our full dataset contained 45,245,582 SNPs. Additional filtering requiring at least 90% data completeness, a minimum quality of 20, SNPs that were biallelic, a minor allele frequency of 0.05, and markers at least 10 kilobases (kb) apart to generate the final ingroup VCF produced 15,368 SNPs from 25 *P. modestum* individuals for population genomic analysis. The sliding-window dataset of variant and invariant sites contained 1,540,051,645 sites. After captus assembly, we recovered an average of 4,986 tetrapod ultra-conserved element (UCE) loci per sample with an average depth of 8.4 (Table S3).

### Population Structure and History

**Fig. 2.**
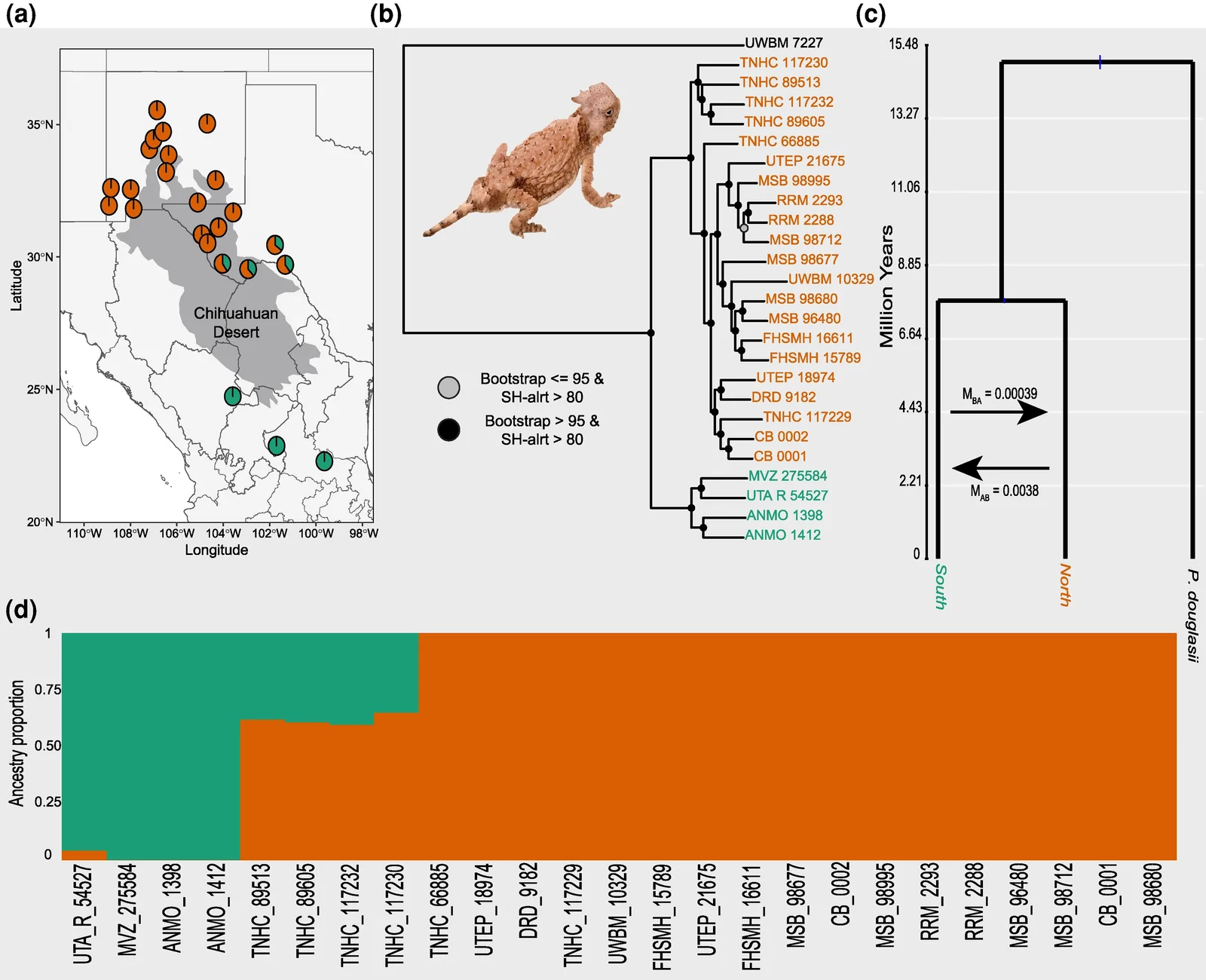
a and d) ADMIXTURE results clustering samples into two populations. Each pie chart and vertical bar represents an individual (in a and d, respectively) with the proportion of each color indicating the proportion of ancestry from a particular cluster. b) A maximum likelihood genealogy inferred using a partitioned analysis in IQ-TREE. Node values are represented with colored dots indicating the ultrafast bootstrap support and Shimoda–Hasegawa approximate likelihood ratio tests (SH-aLRTs). Black dots indicate ultrafast bootstrap support over 95, while gray indicates less than or equal to 95. Both colors indicate SH-aLRTs values above 80. c) Divergence time and migration rate estimates from BPP MSC-M analysis.

Cross-validation error in ADMIXTURE was minimized at *K* = 2 (Fig. S1), yielding two major genetic clusters within *P. modestum*. The clusters corresponded to a North population (Lineage A) spanning New Mexico and Texas and a South population (Lineage B) in Mexico (Fig. 2a). All samples at the geographic extremes of the North and South populations displayed low levels of admixture. Four individuals in western Texas displayed mixed ancestry, where the proportion of ancestry from each population increased with proximity to either cluster (Fig. 2a and d). We also analyzed results for *K* = 3, which split the four admixed individuals in western Texas into their own cluster (Fig. S2a and c).

Our ML phylogenetic analysis of the UCE loci strongly supported two lineages corresponding to the ADMIXTURE populations (Fig. 2b). The four western Texas admixed individuals formed a divergent cluster but were ultimately placed within the North population lineage, concordant with their ancestry makeup. ModelFinder identified 189 partitions, with an average of 761 parsimony informative sites and 7,910,704 total sites. The ML phylogenetic analysis of the mitogenome sequences was concordant with the UCE loci, supporting two lineages corresponding to the ADMIXTURE populations. The mitogenome ML phylogenetic analysis identified three partitions with an average of 479 parsimony informative sites and 15,485 total sites.

Convergence and mixing of the four BPP multispecies coalescent with migration (MSC-M) runs were adequate based on trace plots and ESS values (> 200). Contemporary population sizes for the two lineages were similar and larger than the ancestral size (Table S4). The two lineages diverged approximately 7 million years ago (Ma) (Fig. 2c). The calculated substitution rate for the UCE loci was 0.0004 substitutions per site per million years, approximately half the rate as for fourfold degenerate sites and estimates from GBS data (Perry et al. 2018; Pavón-Vázquez et al. 2024). To accommodate uncertainty in divergence time estimates, we recalculated rates and times using the 95% HPD for the respective node from Leaché and Linkem (2015). Assuming a 12 Ma split between *P. modestum* and *P. douglasii* resulted in a substitution rate of 0.0005 substitutions per site per million years and a North–South split of 6.23 Ma. Assuming a 20 Ma *P. modestum–P. douglasii* split yielded a rate of 0.0003 substitutions per site per million years and a divergence time of 10.38 Ma. The number of migrants per generation was ≪1 for each comparison. However, estimated values were 10× higher from Lineage A (North) to Lineage B (South) than in the opposite direction (Fig. 2c). BPP MSC analyses without gene flow converged (ESS > 200) and yielded very similar parameter estimates to the model with migration (Table S5). Bayes factor calculations indicated significant gene flow from North to South, but not in the opposite direction. Results were identical using prior tail probabilities of both 0.1% and 0.5% for epsilon (ε). The calculated genealogical divergence index (*gdi)* for Lineage A was 0.397, and the *gdi* for Lineage B was 0.443, both falling within the broad zone of uncertainty.

### Intraspecific Variation

We found that the North population had an average π of 0.006 and the South had an average π of 0.009. F_ST_ between the two populations was 0.08. Additionally, a statistically significant correlation between geographic and genetic distance was found, supporting a pattern of isolation-by-distance (IBD) in our dataset (*P*-value < 0.001; rho 0.43).

### Genetic Divergence

**Fig. 3.**
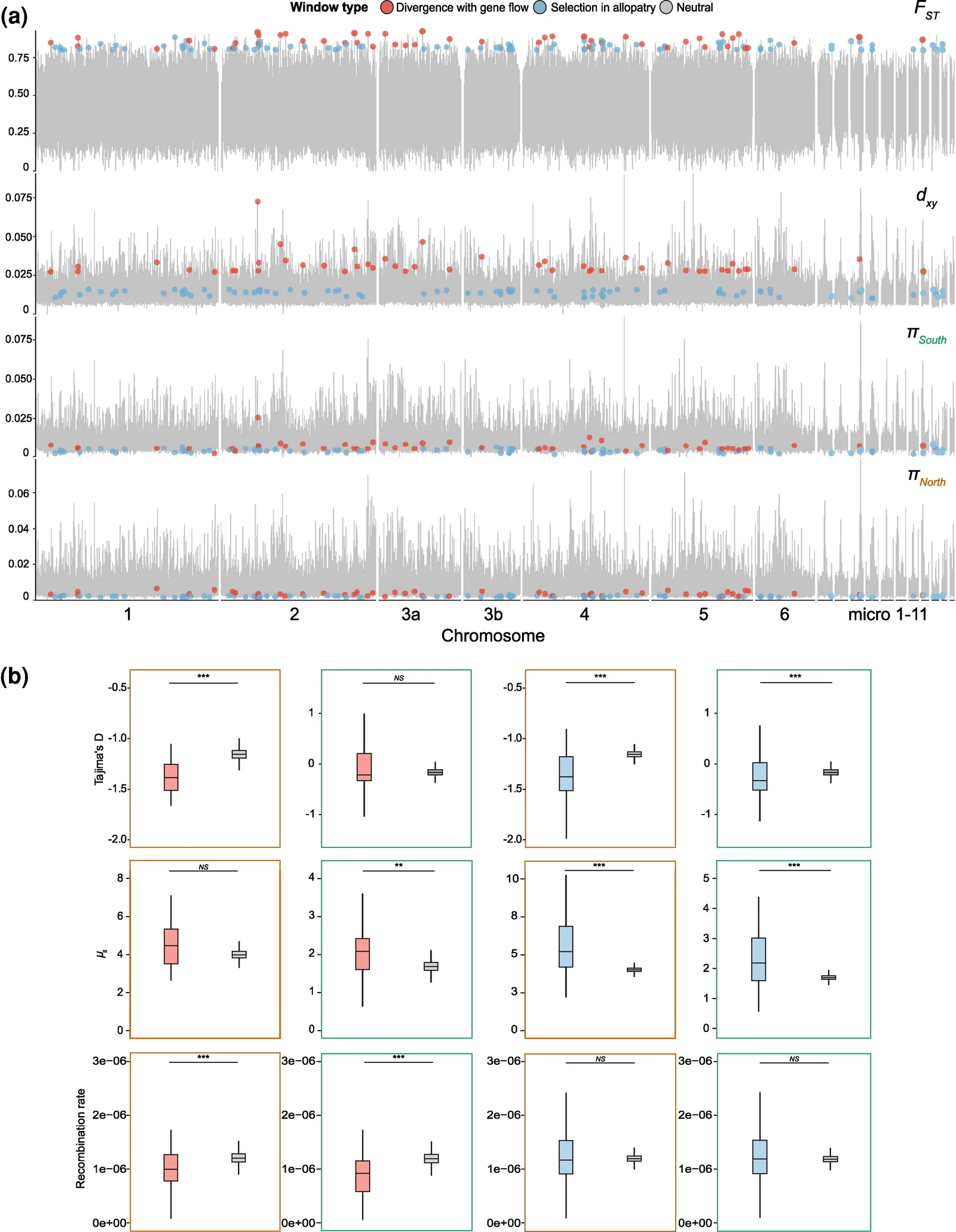
a) Sliding-window divergence and diversity statistics from pixy visualizing genomic divergence islands in divergence with gene flow (red dots) and selection in allopatry (blue dots) scenarios (following Fig. 1) across the genome. Genomic windows that did not follow any of the Fig. 1 scenarios are labeled as neutral and colored gray; the statistic for each row is indicated in the top right. Note that for nucleotide diversity (lower two rows), windows are colored in both populations even though they may only be considered a candidate window in one population (eg Chromosome 2 in the South population). b) Tajima's *D* (top row), *µ_s_* (middle row), and recombination rate estimates (bottom row) for the different scenarios observed in our data (colored boxplots; red = divergence with gene flow; blue = selection in allopatry) versus a π-controlled simulated genomic background (gray boxplots) in each population. The population for each comparison is indicated by the color of the plot outline (North = orange; South = green). Significance is indicated in the above boxes in each plot.

We used relative and absolute metrics of genetic diversity within populations and differentiation between populations to identify non-overlapping 10 kb windows of divergence and their potential drivers (Fig. 3a). In the North population, we found 34 candidate windows containing 13 characterized genes associated with a divergence with gene flow model and 85 candidate windows containing 33 characterized genes associated with a selection in allopatry model (Table S6). In the South population, we found 27 candidate windows containing 11 known genes associated with a divergence with gene flow model and 90 candidate windows containing 30 known genes associated with a selection in allopatry model (Table S6). Twelve candidate windows containing six known genes were linked to a divergence with gene flow model in both populations. No candidate windows were found with characteristics of recurrent selection or geographic sweep before differentiation (Scenarios 3 and 4). Averaging π also identified 34 genomic windows linked to a divergence with gene flow model, and 85 genomic windows associated with a selection in allopatry model, all of which were identified in comparisons without averaging π; therefore, we focused on the within-population π windows. We did not find any directly adjacent candidate windows.

We then compared distributions of Tajima's *D* and the *µ* statistic (here *µ_s_*, to differentiate between µ*_m_*, mutation rate) in candidate windows versus a simulated genomic background as additional tests to understand if candidate windows exhibited patterns that we would expect if they were driven by selection (Fig. 3b). We identified significant differences between Tajima's *D* of divergence with gene flow windows and simulated genomic backgrounds in the North population (Table 1, North: *P*-value = 2.5e-10; South: *P*-value = 0.34) and in both populations (Table 1, North: *P*-value = 2.2e-10; South: *P*-value = 5.5e-6) between selection in allopatry windows and simulated backgrounds using Mann–Whitney *U* tests; Tajima's *D* tended to be lower in outlier windows (Table 1). We also identified significant differences between *µ_s_* of divergence with gene flow windows and simulated genomic backgrounds in the South population (Table 1, North: *P*-value = 0.09; South: *P*-value = 0.001) and in both populations (Table 1, North: *P*-value < 2.2e-16; South: *P*-value = 6.6e-10) between selection in allopatry windows and simulated backgrounds using Mann–Whitney *U* tests; the mean *µ_s_* values were higher in candidate windows than the genomic background (Table 1). We identified 1,945 and 2,441 total candidate outlier *µ_s_* windows in the North and South populations, respectively, and identified overlap between *µ_s_* candidate outliers and Irwin model candidates (Table 1).

**Table 1:** Estimated mean Tajima's *D*, *µ_s_*, and recombination rate in F_ST_ outlier windows for divergence with gene flow (DWGF) and selection in allopatry (SIA) and the number of windows that were identified using both Irwin models and *µ_s_*

	North DWGF	South DWGF	North SIA	South SIA
Tajima's *D*	−1.34 (−1.15)*	−0.12 (−0.16)	−1.33 (−1.15)*	−0.21 (−0.16)*
*µ_s_*	4.80 (4.01)	2.44 (1.70)*	6.00 (4.02)*	2.38 (1.70)*
Recombination rate	1.09e-6 (1.21e-6)*	9.02e-7 (1.19e-6)*	1.30e-6 (1.21e-6)	1.30e-6 (1.19e-6)
Shared candidate outliers (Irwin and *µ_s_*)	3	4	14	17

The mean π-controlled simulated genomic background is indicated in parentheses, and * indicates significant differences between the empirical distribution and simulated distribution.

We tested for differences in the distributions of recombination rate in candidate windows versus a simulated genomic background to contextualize the evolutionary history of candidate windows (Fig. 3b); barriers to gene flow are predicted to accumulate in low recombination regions. We identified significant differences between recombination rate of divergence with gene flow windows and simulated genomic backgrounds in both populations (Table 1, North: *P*-value = 2e-4; South: *P*-value = 2.7e-6) and in neither population (Table 1, North: *P*-value = 0.27; South: *P*-value = 0.73) between selection in allopatry windows and simulated backgrounds using Mann–Whitney *U* tests. The mean recombination rate was lower in divergence with gene flow windows relative to the simulated background (Table 1).

Gene ontology analyses of genes extracted from divergent windows characterized functions of 51 total genes and functional interactions based on gene ontology meta-analyses between seven genes (Tables S6 and S7). For windows in both populations showing signatures of selection in allopatry, a functional interaction was identified concentrated around three F-Box genes: FBXL20 on Chromosome 10 and FBXO11 and MSH6 on Chromosome 1, along with an interaction between KANSL1 on Chromosome 10 and BRD8 on Chromosome 3. Windows of divergence with gene flow in the South population displayed a functional interaction between DHX29 on Chromosome 3 and NOP14 on Chromosome 8. No biological processes were overrepresented in any of our window types.

## Discussion

Allopatric barriers and geographic distance are continually recognized for their role as obstacles to gene flow, promoters of divergence, and in the evolution of reproductive isolation, particularly for species that display restricted home ranges (Munger 1984; Emelianov et al. 2004; Feder et al. 2012; Dool et al. 2022). At first, this divergence and accumulation of barriers to gene flow occurs between populations of the same species; studies of divergence between intraspecific populations are therefore key to understanding the initial stages of speciation (Nosil 2012). In this study, we evaluate whole-genome population genomic data from *P. modestum* to characterize the species' demographic history and understand the drivers of genomic divergence between populations, which may represent the initial stages of speciation. A limited number of genomic regions exhibit patterns of increased F_ST_ and *d_xy_* with reduced π—as expected if they contain genes involved in reproductive isolation—and others display signatures of within-population selection following divergence from the ancestral population, with increased F_ST_ and reduced π. Moreover, recombination rates in these regions follow patterns expected if they are involved in reproductive isolation and within-population selection (Han et al. 2017): the recombination rate is lower in reproductive isolation regions than the genomic background, while the recombination rate was not different in within-population selection regions when compared to the genomic background. These regions represent only a small subset of genomic regions and are not adjacent to each other, suggesting that surrounding regions have not become linked and undergone divergence hitchhiking (Feder et al. 2012). Most of the genome, however, does not follow patterns expected by our models of divergence, underscoring the importance of neutral processes in genomic evolution and highlighting the drawbacks of inferring selective history based on summary statistics alone. We addressed this by performing additional tests for selection. Together, our findings support the role of both neutral and selective processes in shaping genomic divergence and provide insight into the earliest stages of divergence.

### Characterizing Demographic History and Population Structure

Population structure and demographic analyses for *P. modestum* demonstrate a species that, despite maintaining intraspecific genetic diversity through low amounts of gene flow, is composed of two highly divergent lineages in the face of geographic distance. Genomic and mtDNA lineage trees are concordant, suggesting the species has not undergone recent hybridization or introgression events with closely related and geographically concurrent species such as *P. goodei* (Leaché and Linkem 2015; Jezkova et al. 2016).

Demographic analyses indicate that the two lineages, a North and South population, diverged in the Late Miocene, roughly 7 Ma, that populations expanded (relative to the ancestral population), and display low rates of migration based on UCE loci. The divergence time we identified aligns with evidence from other taxa with similar divergence estimates, including *Crotalus scutulatus* and *C. atrox*, along with populations of *Cophosaurus texanus* (Schield et al. 2018; Blair et al. 2026). Our recovered time is also consistent with divergence times of desert tortoises (*Gopherus*) inhabiting other major North American deserts (Edwards et al. 2016). These studies suggest a relatively limited influence of Quaternary glacial–interglacial cycles on lineage divergence. Through using information from a secondary temporal calibration (Leaché and Linkem 2015), we calculate a UCE substitution rate of 0.0004 substitutions per site per million years. This value is approximately half the rate as fourfold degenerate sites (Perry et al. 2018) and rates estimated for GBS/RADseq data (Pavón-Vázquez et al. 2024; Blair et al. 2026). These results support the hypothesis that UCE loci may be under some form of purifying selection that reduces the neutral substitution rate at linked sites. However, we acknowledge that uncertainty in calibration schemes and rates can significantly impact the testing of phylogeographic hypotheses across the Chihuahuan Desert and adjacent regions (Myers et al. 2019).

In addition to shared evidence of older Neogene divergence, the spatial distribution of the two *P. modestum* lineages is broadly consistent with recent genomic studies examining the evolutionary history of the Chihuahuan Desert biota (Schield et al. 2018; Myers et al. 2019; Blair et al. 2026). Because of our sampling constraints and limitations (see below), we are unable to fully disentangle competing hypotheses for the causes of the split. Our results support multiple processes including IBD (Myers et al. 2019; Blair et al. 2026), geomorphological shifts throughout the Central Mexican Plateau (Schield et al. 2018; Blair et al. 2026), and potential vicariance due to the lower Rio Grande (Finger et al. 2022; Blair et al. 2026). The Trans-Pecos region of western Texas also appears to be an area of secondary contact for lineages diverging across ecological gradients (Finger et al. 2022; Blair et al. 2026). Our analyses to detect positive selection across the genome also align with recent hypotheses that divergence across the Chihuahuan Desert region may be reflecting adaptive processes.

Despite this relatively old divergence time between lineages, *gdi* estimates fell into an inconclusive zone, and we therefore cannot recommend a full taxonomic distinction between the two lineages. This uncertainty may be due to a physical gap in our sampling in the region of northern Mexico (Fig. 2a), and greater sampling efforts at the contact zone between the two populations will help determine potential species boundaries between the lineages (Chambers et al. 2025). The extremely low overall rate of migration between these two geographically structured but genetically related populations points to the traditional role assumed by allopatry in decreasing gene flow and promoting the early stages of population divergence (Slatkin 1987; Fitzpatrick et al. 2009).

### Understanding the Landscapes of Diversity and Differentiation

The genomic landscape of diversity and differentiation between the two populations is heterogeneous, underscoring the role of both neutral and selective processes in shaping divergence (Fig. 3). Of the four divergence models in Fig. 1, candidate outlier windows are consistent with selection in allopatry and divergence with gene flow models, with a majority associated with the selection in allopatry model (Fig. 3a). This supports a larger role for within-population selection and potentially local adaptation to divergent environments, which would be unsurprising as *P. modestum* occupies a climatically and geographically heterogeneous range. Putative evidence of local adaptation to environmental conditions has also been identified in other *Phrynosoma* species (*P. cornutum*; Finger et al. 2022: *P. platyrhinos*: Farleigh et al. 2021). However, patterns of population genetic statistics are also impacted by neutral processes, and thus we employ additional tests to determine if patterns may be reflective of adaptive evolution.

Additional investigation of candidate outlier windows provides support for selective processes in generating the peaks of differentiation. Tajima's *D* is lower than the genomic background in all comparisons except for divergence with gene flow windows in the South population, supporting a putative role for selective processes. Tajima's *D*, however, is sensitive to nonselective demographic processes such as population expansion and IBD, which we identified in our data (Tajima 1989). We then estimated the *µ_s_* statistic to detect putative evidence of selective sweeps and found significantly higher *µ_s_* in all our divergence windows except for divergence with gene flow windows in the North population. *µ_s_* outlier windows also overlap with Irwin model windows, particularly in the selection in allopatry model (Table 1). Collectively, this provides multiple lines of evidence for selective processes influencing our outlier windows, but we note that these windows should be treated as candidates for future studies to investigate.

Recombination rate in these windows also aligns with theoretical expectations given each model of divergence identified (Han et al. 2017; Irwin et al. 2018). Windows displaying signs of divergence with gene flow have significantly lower recombination rates than simulated background windows across the genome, following the expectation that barriers to gene flow would evolve in low recombination regions (Han et al. 2017; Martin et al. 2019). Recombination rate estimates at windows classified as selection in allopatry are similar to those of the genomic background, suggesting linked selection is not the driver of these signals and local adaptation may be promoting advantageous combinations of alleles without the effects of recombination (Han et al. 2017).

Gene ontology enrichment analyses of these genes within candidate outlier windows involve various biological processes and identify multiple interaction networks. No particular biological processes were overrepresented, indicating that divergence is not driven by a singular aspect of ecology or life history. Functional interaction networks implicate many cellular processes, including translation initiation, mitosis, and promoting protein stability. In the South population, a functional interaction between DHX29, a translation initiation factor, and NOP14 is identified among potential genes in windows under a divergence with gene flow model (Sweeney et al. 2021). Translation initiation has previously been linked with adaptation to climatic microhabitats in *Anolis* lizards (Akashi et al. 2016). Additionally, a network of F-box proteins, including FBXO11, which has been linked to increased thermal tolerance, is found in shared candidate outlier windows classified under selection in allopatry (Mason and Laman 2020; Luna-Nevárez et al. 2021). Candidate outlier windows classified under a selection in allopatry model in both populations also contain a functional interaction involving BRD8, which has been shown to promote transcription initiation and is also differentiated based on F_ST_ peaks between two tortoise species in the late stages of speciation inhabiting a similar geographic region (Mellor et al. 2025).

The majority of the genome, however, does not display high levels of differentiation. Most of the genomic windows are not classified as candidate outlier windows following any of the conservative summary statistic thresholds we chose based on Irwin et al. (2018) models or identified as candidate outlier windows using the *µ_s_* statistic. This suggests that neutral processes are influencing a large portion of the genome, while those regions influenced by selective processes are small. However, we note that increased sample size could increase statistical power in our selection tests and that relaxing our thresholds would likely result in the detection of additional candidate windows. These results are perhaps unsurprising given our *gdi* results that suggest our samples represent two populations within the same species. At the beginning of speciation, we expect divergence to occur in small regions of punctuated divergence that become linked to other regions of divergence, until the entire genome becomes a barrier to gene flow (Feder et al. 2012). Similarly, our lack of adjacent candidate windows of divergence suggests that these populations are in an early stage of divergence. Altogether, our results suggest that most of the genome is influenced by neutral processes but that many divergence peaks may be primarily driven by selective processes.

### Future Directions and Limitations

While the population structure and demographic history of *P. modestum* characterized here present an important addition to the evolutionary history of phrynosomatid lizards, it is important to recognize the gap in sampling within our dataset, as we were only able to sample individuals from part of the species range (Fig. S2). Some of the effects of this gap may be offset by the high coverage and number of loci recovered, in addition to the sampling distribution relative to the species range (Leaché 2009; Camargo et al. 2010). However, the addition of more individuals increases the accuracy of phylogenetic reconstruction more than the addition of more loci (Maddison and Knowles 2006), and a broader resampling effort that includes regions omitted here may reveal further trends of migration or admixture between the two identified populations and provide greater opportunity to investigate the influences of IBD. Our tests of IBD may have been biased since we lack a continuous sampling and could not test for a gradual increase in genomic differentiation with geographic distance. Furthermore, although whole-genome sequencing of approximately 10× coverage provides a large set of SNPs, the stringent filtering parameters applied reduces our dataset. Deeper coverage, along with alternative tests of selection as applied here, may be necessary for studies attempting to detect selective histories. Incorporation of morphological data to investigate correlations between genetic and phenotypic divergence will also further our understanding of adaptation and potential speciation (Ogden and Thorpe 2002; McGreevy et al. 2024). Despite these limitations, our investigation of genomic divergence and demography within *P. modestum* presents the system as one that can improve our understanding of the relative bounds of speciation.

## Methods

### Data Collection

We obtained data from 26 *Phrynosoma modestum* individuals and one *Phrynosoma douglasii* individual with geographic coordinate data (Table S1). Tissue samples were obtained from museums and field sampling of the Southwestern United States and Mexico. All new field work was conducted under IACUC approval from Brooklyn College (Protocol # 312) and a permit from the New Mexico Department of Game and Fish (#3753). The *P. douglasii* individual was included as an outgroup. We extracted DNA from samples using a Zymo Quick-DNA Miniprep Kit (Zymo Research) following manufacturer's protocols. DNA quantity was measured using a Qubit 4.0 fluorometer (Invitrogen) following manufacturer's recommendations. DNA quality was assessed by running 3 ul of DNA extract on a 1% agarose gel stained with SYBR Green (Thermo Fisher Scientific). Samples that passed QC were sent to Novogene for whole-genome library preparation and sequencing.

### Data Sequencing, Assembly, and Filtering

Novogene generated whole-genome data on an Illumina NovaSeq6000 using 150 bp paired-end sequencing for 27 individuals (26 *P. modestum*, 1 *P. douglasii*) and performed initial trimming and quality control analyses. Sequence data were mapped to the publicly available reference genome of *P. platyrhinos* (Koochekian et al. 2022) using the Burrows–Wheeler Aligner (BWA; Li and Durbin 2009), and SNPs were called and filtered using SAMtools (samtools -C 50-mpileup -m2-F 0.002 -d 1000) (Li et al. 2009) and GATK v4.6.1.0 (DePristo et al. 2011) following the best practices workflow (Van der Auwera and O’Connor 2020). We applied additional filters for population genetic analyses with VCFtools v0.1.16 (Danecek et al. 2011), requiring at least 90% data, a minimum quality score of 20, SNPs that were biallelic, and a minor allele frequency of 0.05. SNPs were thinned by 10 kb to minimize linkage disequilibrium. One ingroup individual was removed due to high levels of missingness that violated filtering (see Table S2).

We performed additional mapping, SNP calling, and filtering for sliding-window analyses since *d_xy_* and π calculations require both invariant and variant sites (Korunes and Samuk 2021). We followed a similar pipeline to Novogene of mapping with BWA but performed SNP calling in BCFtools v1.21 (Danecek et al. 2021) and did not apply a minor allele frequency or minimum allele filters to retain invariant sites. Finally, we used the captus v1.1.0 pipeline (Ortiz et al. 2026) to assemble sequence reads into loci. We followed the captus tutorial (https://edgardomortiz.github.io/captus.docs/) and extracted tetrapod UCEs (5,472 baits for 5,060 loci; Tetrapods-UCE-5Kv1 from https://www.ultraconserved.org/; Faircloth et al. 2012) to represent our loci. We used default settings for each step, except for assembly, where we required that contigs had a minimum depth of 5. We chose the bait with the least missing data if multiple baits were identified for a single locus. We also used captus to extract the mitochondrial genome (mitogenome) using the *Phrynosoma blainvillii* mitogenome (NCBI RefSeq: NC_036492.1) as the reference (Ayala et al. 2017).

### Population Structure and Demographic History

We inferred the number of genetic clusters (*K*) and ancestry of each *P. modestum* sample using ADMIXTURE v1.3.0 (Alexander et al. 2009). ADMIXTURE input files were generated from our filtered ingroup VCF using PLINK v1.9.0b.7.7 (Purcell et al. 2007), which provided us with the input bed file. We determined the best *K* for our dataset by calculating the cross-validation error for *K* values ranging from 1 to 8 and selecting the *K* with the lowest error (Fig. S1). We generated ancestry plots using our optimal *K* of 2 and a *K* of 3 in PopGenHelpR v1.3.2 (Farleigh et al. 2026).

We used IQ-TREE v2.2.2.6 (Minh et al. 2020) to run a partitioned ML phylogenetic analysis on the tetrapod UCE loci from the captus assembly, rooted using *P. douglasii*. ModelFinder (Kalyaanamoorthy et al. 2017) was used to find the best substitution model for each partition using the Bayesian information criterion (BIC) and to merge partitions (MFP + MERGE command) if it improved the overall model fit; initially, each UCE locus was considered a partition. ModelFinder uses a greedy algorithm that first determines the best fit substitution model for individual partitions and then tests whether merging two partitions and assigning them the same substitution model improves the overall model fit (Lanfear et al. 2012; Minh et al. 2020). We evaluated branch support using ultrafast bootstrapping implemented by UFBoot2 (Hoang et al. 2018) and nonparametric Shimoda–Hasegawa approximate likelihood ratio tests (SH-aLRTs) using 10,000 ultrafast bootstraps and 1,000 SH-aLRTs. We also ran a partitioned ML analysis of mitogenomes with IQ-TREE v2.2.2.6 using the same parameters as the UCE dataset (ModelFinder, 10,000 UFboostraps; 1,000 SH-aLRTS) and defining initial partitions by gene.

Finally, we ran a fixed species delimitation and species tree analysis (A00) in BPP v4.8.4 (Flouri et al. 2018) to estimate the demographic history of *P. modestum*. We used the recently developed MSC-M model to estimate population sizes (θ) for both ancestral and current populations, along with divergence times (τ) and effective migration rates (ϖ) between populations (Flouri et al. 2023). Note that BPP estimates mutation-scaled migration rates with ϖ = m/μ*_m_*. Analyses were performed with inverse gamma priors for θ ∼ *IG*(3, 0.004) and τ ∼ *IG*(3, 0.04) and a gamma prior for ϖ ∼ *G*(2, 1). We specified two migration bands, one from Lineage A (North) to Lineage B (South) and vice versa. We used a total of 4,961 UCE loci from all samples in each population and one outgroup sample and performed four independent runs to ensure convergence and adequate mixing. Runs used a burn-in of 50,000, a sample frequency of 2, and 100,000 total samples. Tracer v1.7.2 (Rambaut et al. 2018) was used to assess convergence, mixing, and ESS values (target >200) and also to combine results across runs. To convert ϖ to number of migrants per generation (*M*), we used the following equation:


$$M_{\mathrm{AB}}=(\varpi_{\mathrm{AB}}\;*\;\theta_{B})/4$$

BPP estimates divergence times in units of expected substitutions per site. To convert τs to absolute time in millions of years, we first assumed a divergence time of 15 Ma between *P. modestum* and *P. douglasii* based on the results of a previous phylogenomic study (Leaché and Linkem 2015). We then used the formula μ*_m_* = τ/T to calculate a substitution rate for our UCE loci. Using the calculated μ*_m_*, we rescaled the remaining τ to millions of years. We repeated the analysis under a MSC model without gene flow to compare parameter estimates to results that accommodated migration. The same number of runs, priors, and parameter settings were used. Finally, we calculated Bayes factors (BF_10_) in favor of gene flow (H_1_) versus no gene flow (H_0_) using the Savage–Dickey density ratio following Pavón-Vázquez et al. (2024) and Ji et al. (2023). Calculations were done using a custom python script after combining the Markov chain Monte Carlo (MCMC) files from the four independent runs under the MSC-M model. Tests were done independently for migration in both directions (North -> South, South -> North). We followed previous recommendations and used a BF_10_ cutoff of 20 to indicate strong support for H_1_. Calculations used two values for ε (0.04540202, 0.10349455), which encompassed prior tail probabilities of 0.1% and 0.5%, respectively. These threshold values represent the null hypothesis of no gene flow given the *G*(2,1) prior and were calculated in R.

To provide further support for the independence of lineages and account for potential gene flow, we calculated the genealogical divergence index (*gdi*; Jackson et al. 2017) following Pavón-Vázquez et al. (2024). The *gdi* accounts for bidirectional migration rates and coalescent time to provide a metric of genetic divergence. Specifically, we used BPP to simulate 1 million gene trees with two species (A and B) and three alleles (a_1_, a_2_, b) using demographic parameters estimated from our combined MSC-M runs of the empirical data. We calculated *gdi* as the proportion of simulated gene trees with topology G_x_ = ((a_1_, a_2_), b) where the coalescence time of a_1_ and a_2_ was more recent than the population divergence time τ (Kornai et al. 2024). Two sets of simulations were performed to calculate *gdi* for both lineages. We followed recent guidelines for interpretation of *gdi* values, with estimates ≤ 0.3 indicating conspecificity, values ≥ 0.7 suggesting different species, and intermediate values inconclusive (Jackson et al. 2017; Chambers et al. 2025).

### Intraspecific Variation

We estimated genetic differentiation in our data using PopGenHelpR v1.3.2 (Farleigh et al. 2026). The genetic differentiation between populations (F_ST_) was calculated using the *Differentiation* function. We estimated genetic diversity by calculating the average nucleotide diversity within each population using pixy v1.2.11.beta1 (Korunes and Samuk 2021).

We also evaluated the correlation between geographic and genetic distance to test for evidence of isolation-by-distance (IBD) in our dataset using the R packages nlme v3.1-165 (Pinheiro et al. 2012) and corMLPE v0.0.3 (Pope 2021). These packages use Clarke's ML population effects model to identify a correlation between genetic and geographic distance (Clarke et al. 2002).

### Population Divergence

We generated sliding-window summary statistics of genomic diversity and differentiation in pixy v1.2.11.beta1 (Korunes and Samuk 2021) to determine whether candidate outlier loci may be under selection following the conceptual models of Irwin et al. (2018). For every non-overlapping 10 kb window across the *P. modestum* genome, we calculated nucleotide diversity within both populations (π), along with absolute nucleotide divergence (*d_xy_*) and relative genetic differentiation between populations (F_ST_).

To make comparisons between genomic windows, we standardized per-window diversity metrics by calculating z-scores for each metric. We identified F_ST_ peaks as windows with a z-score of three or above, representing roughly 0.13% of all window F_ST_ scores (Stone and Wessinger 2024). We then classified F_ST_ peaks into different models of divergence using numerical thresholds for standardized π and *d_xy_* z-scores; thresholds were chosen as quantitative cutoffs that conservatively mimic the qualitative patterns outlined in Irwin et al. (2018) (Fig. 1). F_ST_ peaks were classified as divergence with gene flow if the *d_xy_* z-score was three or above and if π was between −0.67 and −3. F_ST_ peaks were classified as selection in allopatry if the *d_xy_* z-score was between −0.67 and 0.67 and if π was between −0.67 and −3. We also used z-scores to classify any F_ST_ peaks as recurrent selection or geographic sweep before differentiation model; no peaks followed these expectations (Fig. 1). A z-score is standardized and a value of 0 indicates that a window is the same as the genome-wide average. These thresholds are arbitrary and conservative; assuming a normal distribution of z-scores, a z-score above 3 or below −3 corresponds to 0.13% of the distribution, a z-score between −0.67 and −3 corresponds to ∼25% of the distribution, and a z-score between −0.67 and 0.67 corresponds to ∼50% of the distribution. We chose these thresholds to limit false positives, but this also means that there are likely windows that may be influenced by these models of divergence that we did not detect. We evaluated patterns of π in two ways: first, by calculating measures of π within each population instead of averaging across populations to identify genomic windows exhibiting patterns of different selective models in each population and, second, by averaging π across populations to calculate z-scores and identify outlier windows across populations. We identified regions with an average *d_xy_* as windows with a z-score between −0.67 and 0.67 (see selection in allopatry; Fig. 1). Both invariant and variant sites were used for this analysis (no minor allele frequency filter) since π and *d_xy_* calculations require both types of sites (Korunes and Samuk 2021).

Next, we investigated whether our observed differentiation peaks may be due to selection or demographic processes acting on those regions of the genome. We first calculated Tajima's *D* for each 10 kb window using vcftools v0.1.15. We compared Tajima's *D* values across different selection model types in each population to a π-controlled simulated genomic background to determine whether our empirical Tajima's *D* values were significantly different from what we would expect by chance. Significant differences between empirical and simulated distributions would provide support that either selection, demography, or a combination of the two led to allelic divergence in a particular window type. We generated the genomic background for each comparison by randomly selecting the same number of windows as in our empirical data and then calculated the mean Tajima's *D* of those windows to represent one simulated observation. We repeated this 10,000 times to construct a null distribution for comparison with our empirical distribution using Mann–Whitney *U* tests in R (R Core Team 2023). We controlled for π differences between our empirical data and the genomic background by limiting our potential background to windows with a π z-score within the range of each window type (Fig. 1).

Next, we performed genome scans for evidence of positive selection by calculating *µ_s_* as implemented in RAiSD v2.9 (Alachiotis and Pavlidis 2018). RAiSD identifies selective sweeps within a population, therefore allowing us to disentangle whether our empirical patterns are driven by selection or demography. RAiSD works by identifying regions with reductions in local polymorphism, shifts in the site frequency spectrum, and high localized linkage disequilibrium (Alachiotis and Pavlidis 2018). RAiSD then uses these patterns to calculate *µ_s_*, where a higher value indicates greater evidence of positive selection. We calculated *µ_s_* in 10 kb sliding windows for both the North and South populations and tested for evidence of selection in two ways. First, we performed the same permutation test as Tajima's *D* but used *µ_s_* instead to understand if each of the divergence window types identified in both populations had a greater *µ_s_* than the genomic background, which would provide support that selection may be affecting those regions. Second, we calculated a z-score for each *µ_s_* window and identified putative candidate regions under selection as windows with a z-score greater than or equal to 3.

### Recombination Rate

We estimated recombination rates in *P. modestum* using pyrho v0.2.0, which uses a composite likelihood approach to account for demographic history when inferring recombination (Kamm et al. 2016; Spence and Song 2019). We estimated demographic history with SMC++ v1.15.2 (Terhorst et al. 2017) with five individuals randomly selected from a single clade of the North population to avoid potential Wahlund effects that could bias recombination rate estimates. We then used the pyrho *lookup* function to generate a lookup table based on sample size and inferred demographic history. Next, we used the *hyperparam* function to evaluate the fit of different block penalty and window size hyperparameters to the data. Finally, we estimated recombination rates using the *optimize* function under a block penalty of 20 and window size of 50 and scaled estimates using a mutation rate of 7.7e-10 (substitutions per site per year: Perry et al. 2018). We then set any recombination rate estimates above the genome-wide 99th percentile to missing to avoid unrealistically high estimates. We then compared the recombination rate in each of the window types to a π-controlled genomic background to understand the role of recombination in generating our observed peaks and the potential impacts of linked selection in reducing *d_xy_* in the selection in allopatry windows. We used the same permutation test procedure outlined above to simulate background genomic recombination rates.

### Gene Ontology

We extracted known genes within highly differentiated windows of the *P. modestum* genome using bedtools v2.31.10 (Quinlan and Hall 2010) to intersect genomic windows of divergence in *P. modestum* with the annotated *P. platyrhinos* genome (Koochekian et al. 2022). We performed a nucleotide BLAST with *Sceloporus undulatus* as the reference database to identify common gene names (Altschul et al. 1990). *Sceloporus undulatus* was chosen because it was the closest taxonomic relation with well-notated BLAST entries (Westfall et al. 2021). STRING v12.0 (Szklarczyk et al. 2025) was then used to understand potential functions and associations between known genes in windows of divergence (Table S7). Our STRING analysis used *Anolis carolinensis* as a reference because its molecular pathways are particularly well-characterized due to its use as a model organism in studies of reptile evolution and development (Eckalbar et al. 2013). We also used Panther v19.0 (Mi et al. 2019) to identify any overrepresented biological process that genes in windows of divergence were involved in. We set *A. carolinensis* as the reference organism and performed a Fisher's exact test with false discovery rate correction for the overrepresentation analysis.

## Supplementary Material

evag217_Supplementary_Data

## Data Availability

Code and scripts to perform analyses are available on GitHub at https://github.com/kfarleigh/modestum_divergence_wgs. Sequencing data are available as NCBI SRA BioProject PRJNA1395130.
