# Supplementary material for: Understanding Genomic Landscapes of Differentiation in Round-Tailed Horned Lizards (Phrynosoma modestum)
Source: Genome Biol Evol. 2026 Aug 28;18(9):evag217. doi: 10.1093/gbe/evag217 (PMC13554808; doi:10.1093/gbe/evag217)
Supplement: Supplementary file 1 [file evag217_supplementary_data.docx]

**Supplemental Materials**

**Table S1**. Description of sample areas for *Phrynosoma modestum* individuals, * indicates outgroup *Phrynosoma douglasii* individual.

| **Sample** | **Locality** | **Country** | **State** | **County** | **Latitude (N)** | **Longitude (W)** |
| --- | --- | --- | --- | --- | --- | --- |
| ANMO_1398 | Carr. Aldama-Cuencame | Mexico | Durango |  | 24° 44' 20.1984" | -103° 35' 18.3978" |
| ANMO_1412 | Carr. Aldama-Cuencame | Mexico | Durango |  | 24° 44' 20.1984" | -103° 35' 18.3978" |
| CB_0001 | Santa Rosa Lake State Park | USA | New Mexico | Guadalupe | 35° 2' 6.5034" | -104° 41' 20.328" |
| CB_0002 | Karr Ranch Rd | USA | New Mexico | Eddy | 32° 53' 58.2" | -104° 19' 37.5234" |
| DRD_9182 | Kent | USA | Texas | Culberson | 31° 7' 15.708" | -104° 12' 23.4354" |
| FHSMH_15789 |  | USA | New Mexico | Hidalgo | 31° 56' 16.8894" | -108° 55' 28.614" |
| FHSMH_16611 |  | USA | New Mexico | Grant | 32° 33' 16.3944" | -107° 59' 22.095" |
| MSB_96480 | NE Foothills of Ladrone Mt | USA | New Mexico | Socorro | 34° 26' 50.7654" | -107° 0' 0" |
| MSB_98677 | White Rock Canyon Rd | USA | New Mexico | Hidalgo | 32° 36' 22.5354" | -108° 50' 25.9794" |
| MSB_98680 | Dragon's Back Mountain Bike Trail | USA | New Mexico | Sandoval | 35° 32' 17.2926" | -106° 51' 9.9498" |
| MSB_98712 | Rio del Oro Loop Rd | USA | New Mexico | Valencia | 34° 43' 41.2314" | -106° 35' 38.184" |
| MSB_98995 | White Sands Missile Range | USA | New Mexico | Sierra | 33° 12' 5.9076" | -106° 27' 47.0304" |
| MVZ_275584 | SW of Santo Domingo-Salinas Hwy | Mexico | San Luis Potosi |  | 22° 52' 17.04" | -101° 42' 1.764" |
| RRM_2081 | W side of Hueco Mts | USA | Texas | El Paso | 31° 42' 36.4782" | -105° 59' 49.7616" |
| RRM_2288 | S of Magdalena | USA | New Mexico | Socorro | 34° 4' 21.8964" | -107° 11' 13.8294" |
| RRM_2293 | Foothills of Sierra Oscurra | USA | New Mexico | Socorro | 33° 50' 56.3562" | -106° 21' 16.635" |
| TNHC_117229 | Meteor Rd | USA | Texas | Loving | 31° 40' 55.02" | -103° 33' 52.128" |
| TNHC_117230 | Co Rd 169 | USA | Texas | Presidio | 29° 44' 42.72" | -104° 1' 59.772" |
| TNHC_117232 | Independence Creek Rd | USA | Texas | Terrell | 30° 27' 50.1228" | -101° 45' 59.67" |
| TNHC_66885 | Sierra Vieja Mts, Vieja Pass | USA | Texas | Presidio | 30° 32' 51.561" | -104° 40' 46.2066" |
| TNHC_89513 | FM 2627 | USA | Texas | Presidio | 29° 32' 15.108" | -102° 55' 37.956" |
| TNHC_89605 | Pandale | USA | Texas | Val Verde | 29° 42' 18.396" | -101° 19' 32.124" |
| UTA_R-54527 | Carretera Ciudad del Maiz-Rayón | Mexico | San Luis Potosi |  | 22° 17' 20.2914" | -99° 38' 18.9954" |
| UTEP_18974 | Creek feeding into Green River Tank | USA | Texas | Hudspeth | 30° 50' 38.76" | -104° 56' 0.837" |
| UTEP_21675 | Hwy 506 | USA | New Mexico | Otero | 32° 3' 20.8794" | -105° 5' 57.2994" |
| UWBM_10329 | E of Hermanas on Hwy 9 | USA | New Mexico | Luna | 31° 49' 21.7518" | -107° 51' 46.4826" |
| UWBM_7227* | Whiskey Dick Wildlife Area | USA | Washington | Kittias | 46° 57' 36" | -120° 8' 24" |

**Table S2**. Description of sequencing statistics for each sample included in this study.

| **Sample** | **Total reads** | **Mapped reads** | **Mapping rate (%)** | **Mean Depth** |
| --- | --- | --- | --- | --- |
| ANMO_1398 | 156,840,714 | 147,530,115 | 94.06 | 10.49 |
| ANMO_1412 | 184,775,576 | 173,580,418 | 93.94 | 12.43 |
| CB_0001 | 170,056,600 | 160,173,985 | 94.19 | 11.25 |
| CB_0002 | 1561,685,84 | 147,168,023 | 94.24 | 10.3 |
| DRD_9182 | 216,434,726 | 203,181,419 | 93.88 | 14.27 |
| FHSMH_15789 | 124,752,768 | 116,221,604 | 93.16 | 8.39 |
| FHSMH_16611 | 136,770,378 | 128,745,895 | 94.13 | 9.23 |
| MSB_96480 | 165,605,308 | 155,094,579 | 93.65 | 11.07 |
| MSB_98677 | 167,583,434 | 157,633,034 | 94.06 | 11.08 |
| MSB_98680 | 144,175,988 | 135,477,985 | 93.97 | 9.81 |
| MSB_98712 | 154,081,250 | 144,378,927 | 93.7 | 10.87 |
| MSB_98995 | 146,676,728 | 137,507,279 | 93.75 | 10.08 |
| MVZ_275584 | 168,625,166 | 156,910,490 | 93.05 | 10.95 |
| RRM_2081 | 116,843,970 | 31,610,765 | 27.05 | 9.97 |
| RRM_2288 | 139,379,022 | 126,885,046 | 91.04 | 7.73 |
| RRM_2293 | 101,982,616 | 94,767,735 | 92.93 | 7.26 |
| TNHC_117229 | 615,42,144 | 57,473,958 | 93.39 | 4.66 |
| TNHC_117230 | 162,635,040 | 152,713,095 | 93.9 | 11.24 |
| TNHC_117232 | 146,416,076 | 137,718,557 | 94.06 | 10.21 |
| TNHC_66885 | 165,982,074 | 155,581,482 | 93.78 | 11.19 |
| TNHC_89513 | 166,913,824 | 156,592,293 | 93.82 | 11.71 |
| TNHC_89605 | 141,893,238 | 133,213,806 | 93.88 | 9.79 |
| UTA_R-54527 | 171,487,628 | 161,438,404 | 94.14 | 11.61 |
| UTEP_18974 | 105,512,680 | 98,440,779 | 93.3 | 7.46 |
| UTEP_21675 | 97,981,490 | 90,934,861 | 92.81 | 6.82 |
| UWBM_10329 | 149,387,068 | 140,549,550 | 94.08 | 9.86 |
| UWBM_7227 | 172,797,520 | 158,880,949 | 91.95 | 11.4 |

**Table S3**. Captus assembly statistics for each sample included in this study.

| **Sample** | **Assembled contigs** | **Mean Depth** | **Extracted loci** | **% genes recovered** |
| --- | --- | --- | --- | --- |
| ANMO_1398 | 1,189,176 | 7.70 | 5,090 | 93.0 |
| ANMO_1412 | 1,166,892 | 8.59 | 5,092 | 93.1 |
| CB_0001 | 809,073 | 9.83 | 5,093 | 93.1 |
| CB_0002 | 861,544 | 9.27 | 5,090 | 93.0 |
| DRD_9182 | 1,027,038 | 9.99 | 5,099 | 93.2 |
| FHSMH_15789 | 1,253,903 | 8.27 | 5,063 | 92.5 |
| FHSMH_16611 | 905,106 | 8.64 | 5,092 | 93.1 |
| MSB_96480 | 951,329 | 9.25 | 5,032 | 92.0 |
| MSB_98677 | 809,992 | 10.00 | 5,092 | 93.1 |
| MSB_98680 | 831,588 | 8.96 | 5,088 | 93.0 |
| MSB_98712 | 819,818 | 9.84 | 5,092 | 93.1 |
| MSB_98995 | 815,463 | 8.69 | 5,090 | 93.0 |
| MVZ_275584 | 1,119,828 | 8.70 | 5,097 | 93.1 |
| RRM_2288 | 1,399,739 | 8.59 | 4,937 | 90.2 |
| RRM_2293 | 1,556,661 | 7.47 | 4,901 | 89.6 |
| TNHC_117229 | 1,945,444 | 5.27 | 4,501 | 82.3 |
| TNHC_117230 | 1,023,242 | 8.43 | 5,087 | 93.0 |
| TNHC_117232 | 977,575 | 8.29 | 5,088 | 93.0 |
| TNHC_66885 | 999,003 | 8.65 | 5,091 | 93.1 |
| TNHC_89513 | 994,478 | 9.09 | 5,101 | 93.2 |
| TNHC_89605 | 1,028,500 | 7.98 | 5,091 | 93.0 |
| UTA_R-54527 | 819,571 | 9.13 | 5,101 | 93.2 |
| UTEP_18974 | 1,491,906 | 7.05 | 4,928 | 90.1 |
| UTEP_21675 | 1,820,505 | 7.07 | 4,873 | 89.1 |
| UWBM_10329 | 2,039,949 | 6.23 | 4,020 | 73.5 |
| UWBM_7227 | 2,091,819 | 6.21 | 4,811 | 87.9 |

**Table S4**. Parameter estimates from BPP MSC-M analyses using 4961 UCE loci. Results are from four independent runs. Two migration events were specified in the model. Values of theta and tau are multiplied by 1000. A = North, B = South.

| **Parameter** | **Mean** | **95% HPD lower** | **95% HPD upper** |
| --- | --- | --- | --- |
| 𝜃_A_ | 12.3 | 12.2 | 12.4 |
| 𝜃_B_ | 10.6 | 10.3 | 10.8 |
| 𝜃_AB_ | 3.2784 | 3.184 | 3.368 |
| 𝜃_root_ | 19.6 | 19 | 20.2 |
| 𝜏_root_ | 5.9882 | 5.902 | 6.072 |
| 𝜏_AB_ | 3.1152 | 3.085 | 3.145 |
| $\varpi$_A->B_ | 1.416 | 0.7804 | 2.0779 |
| $\varpi$_B->A_ | 0.1263 | 0.0156 | 0.2483 |

| **Table S5**. Parameter estimates from BPP MSC analyses without migration using 4961 UCE loci. Results are from four independent runs. Values of theta and tau are multiplied by 1000. A = North, B = South. | | | |  |
| --- | --- | --- | --- | --- |
|  |  |  |  |  |
|  |  |  |  |  |
|  |  |  |  |  |
| **Parameter** | **Mean** | **95% HPD lower** | **95% HPD upper** |  |
| 𝜃_A_ | 12.3 | 12.2 | 12.4 |  |
| 𝜃_B_ | 10.6 | 10.4 | 10.9 |  |
| 𝜃_AB_ | 3.3256 | 3.235 | 3.417 |  |
| 𝜃_root_ | 19.5 | 18.9 | 20.1 |  |
| 𝜏_root_ | 5.9988 | 5.916 | 6.088 |  |
| 𝜏_AB_ | 3.086 | 3.056 | 3.113 |  |

**Table S6.** Molecular functions of genes identified within 10kb windows of divergence. * indicates a gene’s presence in a functional interaction network.

| **Gene name** | **Associated function** |
| --- | --- |
| **Divergence with gene flow**  *North Population* |  |
| DAPK1 | Involved in inducing cell death |
| NOA1 | Regulation of mitochondrial protein synthesis and mitochondrial respiratory complexes |
| SH3GLB1 | Involved in coatomer-mediated retrograde transport and support of mitochondrial membrane |
| MAATS1 | Involved in spermatogenesis |
| PLA2G4A | Catalyzes hydrolysis of phospholipids into fatty acids |
| ZC3H12C | Enables RNA endonuclease activity and mRNA binding |
| TEDC1 | Involved in centriole stability and positive regulation of smoothened signalling pathway |
| *South Population* |  |
| NBN | Involved in DNA damage and repair |
| DHX29* | Translation initiation, specifically during initiation codon selection |
| CHL1 | Nervous system functioning and support |
| DOCK1 | Involved in phagocytosis and cell motility |
| PRKAA1 | Involved in phosphorylation regulation |
| *Shared* |  |
| NOP14* | rRNA processing and small ribosomal subunit assembly |
| RIC8B | Promotes binding and folding of G proteins |
| GGH | Hydrolyzes sidechain of pteroylpolyglutamates |
| PRAF2 | Involved in L-glutamate transmembrane transport |
| RGS7 | Regulation of G protein signaling 7 |
| SCAF11 | Enables RNA and protein binding |
| **Selection in allopatry**  *North Population* |  |
| MOCS1 | Involved in activation of molybdenum |
| TMEM132C | Transmembrane protein |
| ERCC6L2 | Promotes DNA repair |
| *Shared* |  |
| FBXL20* | F-box protein, enables protein binding |
| FBXO11* | F-box protein, involved in protein recognition and binding |
| MSH6* | Recognition of mismatched nucleotides for repair |
| KIAA0825 | Enables protein binding |
| KANSL1* | Involved in transcription initiation, enhancer regulation, and mitosis |
| BRD8* | Coactivator in transcription activation, potentially involved in DNA repair |
| TTLL5 | Involved in ligase activity and regulation |
| FAM120C | Transmembrane protein involved in RNA binding |
| TBRG1 | Nucleoplasm protein, can act as growth inhibitor |
| SLC25A12 | Promotes calcium binding in the mitochondria |
| MED23 | Involved in gene transcription activation |
| TAF5L | Involved in myogenic transcription and differentiation |
| SLC37A1 | Transport of glucose products to endoplasmic reticulum |
| DSCAML1 | Involved in cell adhesion by promoting neuronal differentiation |
| U2AF2 | Involved in RNA binding and splicing |
| CEP164 | Primary cilia assembly and maintenance |
| AFF4 | Increases rate of transcription |
| SLC44A1 | Involved in transmembrane transport |
| GGACT | Protein degradation via transglutaminase degradation |
| FAM120A | Enables RNA binding |
| BPTF | Involved in DNA transcription and repair |
| SEC24B | Involved in vesicle trafficking |
| GLOD5 | Protein with a glyoxylase domain |
| HS2ST1 | Involved in signalling of kidney formation |
| B3GLCT | Involved in glucose transfer |
| HSPBAP1 | Involved in cell growth, differentiation, and stress response |
| EPHA7 | Binds ligands involved in brain development |
| MFAP3 | Extracellular protein |
| PSMD14 | Maintains protein homeostasis via removal of damaged or misfolded proteins |
| AHCYL1 | Cellular regulator involved in fluid secretion, mRNA processing, and DNA replication |

**Table S7.** STRING network statistics, reported for interactions between genes identified in divergent windows of each population and under each Irwin scenario. PPI represents protein-protein interaction.

| **STRING statistic** | **North**  **DWGF** | **South**  **DWGF** | **North**  **SIA** | **South**  **SIA** |
| --- | --- | --- | --- | --- |
| Number  of nodes | 13 | 11 | 33 | 30 |
| Number  of edges | 0 | 1 | 3 | 2 |
| Clustering coefficient | 0 | 0.182 | 0.121 | 0.133 |
| PPI  p-value | 1 | 0.333 | 0.378 | 0.276 |

**Figure S1.** Cross-validation error for ADMIXTURE analyses for values of K = 1–8. K = 2 minimizes cross-validation error at 0.434.

**Figure S2**. ADMIXTURE results based on K=3 and a sampling map. A & C) A piechart map and barchart representing the ancestry of each individual with the proportion of each color indicating the proportion of ancestry from a particular cluster. B) A map of our samples (grey points) in relation to the species distribution (light grey) and Chihuahuan desert (dark grey); shapefiles were downloaded from the International Union for the Conservation of Nature and ESRI, respectively.

**Figure S3.** Maximum-likelihood (ML) genealogy of whole mitochondrial DNA (mtDNA) genome sequences using IQ-TREE. Mitochondrial genomes were extracted using captus, with the *Phrynosoma blainvilli* mtDNA genome as the reference. Results are generally concordant with the tetrapod ultra-conserved element genealogy presented in the main text.
